# Access and adequate utilization of malaria control interventions in rural Malawi: a descriptive quantitative study

**DOI:** 10.1186/s12936-018-2253-1

**Published:** 2018-03-06

**Authors:** Alinune Nathanael Kabaghe, Michael Give Chipeta, Robert Sean McCann, Dianne Jean Terlouw, Tinashe Tizifa, Zinenani Truwah, Kamija Samuel Phiri, Michèle van Vugt

**Affiliations:** 1 Center of Tropical Medicine and Travel Medicine, Department of Infectious Diseases, Academic Medical Center, University of Amsterdam, 1105 AZ Amsterdam, The Netherlands; 2 School of Public Health and Family Medicine, College of Medicine, University of Malawi, Blantyre 3, Malawi; 3 Lancaster Medical School, Lancaster University, Lancaster, LA1 4YG UK; 4 Malawi-Liverpool Wellcome Trust Clinical Research Program, Queen Elizabeth Central Hospital, College of Medicine, Blantyre, Malawi; 5 Laboratory of Entomology, Wageningen University and Research Centre, 6708 PB Wageningen, The Netherlands; 6 Management Sciences for Health-Malawi Program, EBC Building, Off Paul Kagame Road, Private Bag 398, Lilongwe 3, Malawi

**Keywords:** Malaria, Intervention, Rural communities, Malawi

## Abstract

**Background:**

Despite the availability of cost effective malaria control interventions, such as insecticide-treated bed nets (ITN), diagnosis and effective treatment of malaria, and intermittent preventive treatment during pregnancy (IPTp), the lack of equitable access and coverage affect utilization of these interventions in rural communities. Aggregated rates of access and utilization of malaria interventions in national surveys mask substantial variations in intervention coverage. Utilization of interventions and factors affecting utilization need investigation in rural communities.

**Methods:**

One year of quantitative data collected from a rolling Malaria Indicator Survey (April 2015–April 2016) in Chikhwawa District, Malawi, before the ITN distribution campaign, were analysed. Univariate analyses were used to quantify rates of ITN usage, care-seeking for fever in children aged 6–59 months and women aged 15–49 years and IPTp uptake (for women aged 15–49 years with a recent delivery). Results were compared to national survey estimates; factors associated with these outcomes were determined using multivariate regression models.

**Results:**

A total of 2046 participants were included from 1328 households; 56.6% were women aged 15–49 years and 43.4% were children aged 6–59 months. Reported ownership of at least one ITN per household and under-five children ITN use the previous night were 35.3 and 33.5% compared to 70.2 and 67.1%, respectively, in the national survey; ITN use was higher in high wealth quintile households than low quintile ones. For participants with recent fever, 37.6 and 19.5% sought care and sought care within 24 h, respectively. Care-seeking was lower for febrile women than febrile children [aOR, 95% CI 0.53 (0.35–0.81)]. Uptake of two and three or more doses of IPTp were 40.6 and 15.0%, respectively, among women with a pregnancy in the last 2 years.

**Conclusion:**

To achieve effective malaria control, fine-scale or district-based surveillance should be used to identify and target communities requiring scaling up of interventions. Qualitative research and a participatory community approach should be used to address behavioural factors affecting how people make use of interventions.

## Background

The World Health Organization’s first aim towards achieving a world free of malaria is to ensure “universal access to malaria prevention, diagnosis and treatment” [1]. Malaria prevention using insecticide-treated bed nets (ITN), and treatment with artemisinin-based combination therapy (ACT), are cost effective, and have reduced disease incidence and prevalence in the African population [2, 3]. For pregnant women, intermittent preventive therapy in pregnancy with sulfadoxine–pyrimethamine (IPTp-SP) is a cost effective intervention to prevent adverse effects of malaria in pregnancy for both women and the unborn child, and reduces disease burden in stable transmission areas [4–7]. However, the lack of equitable access (availability, affordability, and acceptability) [8] and coverage affect utilization of the interventions in poor and rural communities [9, 10]; poor communities have a disproportionately higher burden of malaria infection than wealthier communities [11]. Other key factors affecting utilization depend on the quality of the health system and traditions of behaviour in the community [12].

Although the prevalence of *Plasmodium falciparum* parasitaemia in children under 5 years old in Malawi has declined between 2010 and 2014 according to the 2014 Malawi Malaria Indicator Survey (MMIS), the prevalence is three times higher in rural than urban areas [13]. Given that the burden of malaria is high in rural communities, determinants of community utilization of the efficacious interventions need to be identified and specifically addressed to improve and maximize their effectiveness in malaria control. While national-level surveys provide statistics on the access and utilization of malaria control interventions, these aggregated survey estimates may mask substantial variations in intervention coverage that exist in rural communities [14].

In this study, data from repeated cross-sectional malaria indicator surveys were analysed to quantify access and utilization of adequate prompt diagnosis and treatment of reported fever in children and women in a rural Malawian community. ITN usage (before the ITN distribution campaign), and an evaluation of access to and utilization of adequate IPTp in pregnant women are described and compared to 2014 MIS. Finally, factors associated with ITN and IPTp utilization and care-seeking for fever in the rural community are investigated.

## Methods

### Study design and setting

This paper describes and analyses quantitative data collected during a rolling malaria indicator survey (rMIS) in rural Malawi. rMIS involves a sequence of cross-sectional surveys collecting demographic and health-related data from different sampled households, within a defined community [15]; each cross-sectional survey used a questionnaire and laboratory procedures adapted from standardized Malaria Indicator Survey (MIS) [16].

The study setting has been described previously [17]. Briefly, rMIS was conducted in communities surrounding Majete Wildlife Reserve (MWR) in Chikhwawa district, southern Malawi from April 2015 to April 2016. The study site has been part of the Majete Malaria Project (MMP) since February 2014. Transmission of (mainly falciparum) malaria is stable, peaking during the rainy season (December to May). The population relies mainly on rain-fed subsistence and small-scale commercial farming.

The MMP catchment area has five public and two private health centres, offering primary health-care services. One district hospital, offering both primary and secondary health-care services, is located on average 23 km (range 3–42 km) from the households in the catchment area (by Euclidian distance approximation). There are some private clinics and shops which stock anti-malarial drugs such as sulfadoxine–pyrimethamine and quinine. On average, the number of health workers, defined as nurse or clinician, is 2.1 per 10,000 population in the primary health-care system [18]. Malaria diagnosis using malaria rapid diagnostic test (*SD Bioline malaria Ag Pf* HRP-2 Standard Diagnostics Inc, Korea) and treatment with the recommended anti-malarial drug artemether–lumefantrine (AL), as first line for uncomplicated cases, artesunate–amodiaquine as second line, and intravenous quinine or parenteral artesunate for severe malaria, are freely available for all ages in both public and private health centres. Private clinics and dispensaries do not provide malaria tests although they sell anti-malarial drugs. During the study period, community health workers, locally known as Health Surveillance Assistants, prescribed malaria treatment to children below 5 years based on symptoms only without a confirmatory parasitological test. These community health workers are based in communities defined as “hard to reach” by the Malawi Ministry of Health. Intermittent preventive-treatment in pregnancy with sulfadoxine–pyrimethamine (IPTp-SP) is also free in all facilities. Private facilities charge a small consultation fee for all patients and for drugs other than anti-malarial drugs; diagnosis and treatment for malaria are free in both private and public facilities.

### Participants

Participants were women aged 15–49 years and children aged 6–59 months from sampled households in MWR perimeter. Males above 5 years old were not included as participants. For IPTp-SP utilization, women who were recently pregnant (delivered a live or stillbirth in the preceding 2 years), were included.

### Data collection

An enumeration of all households in the MWR perimeter was conducted from August to December 2014. All household and primary health-care facility Global Positioning System (GPS) coordinates were recorded using a Samsung Galaxy Tab 3 running Android 4.1 Jellybean operating system. Age, sex and relationship to head of household for all household members were also recorded. Household sampling has been described previously [17]. Briefly, from the enumeration database, a probability-based sample of households (without replacement) per survey sequence, were selected using inhibitory geostatistical sampling initially [19], then adaptive geostatistical sampling (AGD) [20]. Inhibitory sampling requires that a pair of sampled units/locations be separated by a specific distance and AGD relies on previous sample results to sample new units/locations. Members of the sampled households, specifically all women aged 15–49 years, and all children aged 6–59 months (accompanied by their guardian), were invited to a central location within the community. The members were requested to bring their health passport, a book containing details of visits to health facilities.

At the central location, 2–4 trained research assistants and one nurse administered an electronic-based questionnaire adapted from the MIS [16]. An informed consent was administered in the local language (Chichewa) to the head of household or any household member above 18 years old. Where available, women aged 15–49 years and/or guardians of children aged 6–59 months from consented households were interviewed regarding household ownership of at least one ITN and use by eligible individuals during the previous night, recent pregnancies (for women) and recent fever (fever in the preceding 2 weeks). For the women aged 15–49 years, highest education attained and details of IPTp-SP utilization in the recent pregnancy (within the preceding 2 years), were recorded. IPTp-SP doses were confirmed in the respondent’s health passport. Socio-demographic details and insecticide-treated bed net ownership and use were recorded for all households.

### Quantitative variables

The primary outcomes, ITN use the previous night and care-seeking for fever by the participants (children aged 6–59 months and women aged 15–49 years), and IPTp-SP utilization by women with a pregnancy in the previous 2 years, were binary. Care-seeking applied to participants who reported a fever within the preceding 2 weeks. ITN utilization is reported based on interview responses. IPTp-SP utilization was reported primarily based on documented proof in health passport, although participant-reported IPTp-SP is also reported. Adequate IPTp-SP use was based on health passport-confirmed three or more doses of SP.

Independent variables were age, wealth quintile, number of occupants in the household, highest education attained (for IPTp-SP), and proximity to health facility. Socio-economic status was derived using principal component analysis [21] and reported as relative wealth quintiles based on ownership of specific household items. Proximity to health facility was calculated based on Euclidian distance between geolocations of each household to its nearest health facility.

### Statistical methods

Stata software version 13 (StataCorp, Texas, USA) was used for statistical analysis. For univariate analysis, means and proportions are reported. T tests and χ^2^ for continuous and binary variables, respectively, were determined using bivariate analysis. Multivariate binomial logistic regression was used to determine factors associated with each of the three separate primary outcomes: ITN use; care-seeking for fever; and confirmed three or more doses of IPTp-SP during pregnancy. The variables included in each model were selected a priori based on whether they can potentially affect the outcome from previous reports. For example, distance and season have been previously reported to affect care seeking in the same population [22, 23]. Only variables with *p* values less than 0.05 were considered statistically significant in the multivariate analysis.

### Ethical consideration

The rMIS study was reviewed and approved by College of Medicine Research and Ethics Committee (P.09/14/1631) in Malawi. All participants or their legal guardians signed an informed consent before enrolment.

## Results

### Summary characteristics of study participants

Out of 1568 households sampled, 1328 (84.7%) were completed; 240 households were not completed due to unavailability of respondents or refusal to participate (Table 1). For household information, we include 1251 households which had eligible children and women. Of 2046 participants, 887 were children and 1159 were women aged 15–49 years. The median age of the children and women were 2.6 and 29.3 years respectively. Fifty-one percent of the women had some primary education; few women had completed secondary school. There were more women with no formal education in the study area compared to the 2014 MMIS [13]. Reported ownership of at least one ITN per household was 35.3% which was lower than the 2014 MMIS. Fewer households had access to electricity, protected drinking water and owned valuable household items in the study population compared to the 2014 MMIS. The mean distance to a health facility was 2.9 km and the average household size was 4.5 people.

**Table 1:** Participants summary characteristics

Summary characteristic	n (%)	MIS 2014 n* (%) [13]
Households	1328	
Participants	2046	
Children	887 (43.4)	
Women	1159 (56.6)	
Highest level of education women (*3 missing*)		N = 2897
No school	382 (33.0)	293 (14.4)
Some primary	590 (51.0)	1763 (62.6))
Completed primary or some secondary	137 (11.9)	742 (20.5)
Completed Secondary or further	47 (4.1)	99 (2.4)
ITN available in household	722 (35.3)	(70.2)

	Median (IQR)	
Median age in years (IQR)		
Children	2.6 (1.6–3.8)	
Women	29.3 (22.2–36.1)	

	Mean, min–max (SD)	
Household size (SD)	4.5 (2.03)	
Distance to a health facility in km, minimum–maximum	2.9, 0.1–7.6	
Household facilities and possessions (N = 1251)	n (%)	(%)
Electricity	29 (2.3)	(11.9)
Radio	494 (39.5)	(52.2)
Mobile phone	496 (39.7)	(48.8)
Television	44 (3.5)	(14.0)
Drinking water		
Piped into dwelling	16 (1.3)	(14.2)
Piped into yard/plot	7 (0.6)	(10.6)
Borehole	1060 (84.7)	(55.8)
Unprotected source (river/surface water)	98 (7.8)	(4.1)
Flush own toilet	14 (1.1)	(3.4)

*ITN* insecticide-treated bed net, *IQR* interquartile range, *SD* standard deviation

n* The total is included where provided

### ITN use, care-seeking, diagnosis, and treatment of fever

Overall, reported ITN use in the previous night was 31.6%; there was no difference in use between women aged 15–49 years old and children aged 6–59 months old. ITN use in children aged 6–59 months was 33.5% and lower than 2014 MMIS estimate of 67.1% [13]. In the study, 401 (19.6%) participants reported fever within the preceding 2 weeks of the surveys (Table 2). By age categories, more children (22.1%) than women (17.6%) had recent a fever (*p* = 0.013). Although 37.6% of those with a recent fever sought treatment at a facility providing diagnosis and treatment of malaria, only 19.5% sought it within 24 h. In total, 13.0% bought drugs or presented to a private clinic. The average minimum distance travelled by children and women were 3.0 and 2.7, respectively, and not different statistically (*p* = 0.487). Regarding malaria diagnosis and treatment, a finger prick for a malaria test was reported in 41.1% which is higher than reported in the 2014 MMIS (32.4%) [13]; children were more likely than women to receive a finger prick. Although the outcome of the malaria test was unknown, 5.5% of the participants took non-recommended first-line anti-malarial drugs. There were 20.9 and 22.0% children and women, respectively, who paid for health services.

**Table 2:** ITN use, care-seeking, diagnosis and treatment of fever

Participants	Total	Children^a^ (%)	Women^a^ (%)	*p* value
ITN used previous night	646 (31.6)	297 (33.5)	349 (30.2)	0.104

Reported fever	N (%)	n (%)	n (%)	*p* value
Total with fever (% of participants)	401 (19.5)	196 (22.1)	205 (17.6)	0.013*
Sought treatment at PHC	152 (37.6)	88 (44.9)^b^	64 (31.2)	0.005*
Sought treatment at PHC within 24 h	78 (19.5)	43 (21.9)	35 (17.1)	0.219*
Self-treatment or private clinics	52 (13.0%)	26 (13.2)	26 (12.7)	0.862*
Mean distance to access care in km, (95% CI)				
PHC		3.0, (2.5–3.4)	2.7, (2.0–3.3)	0.487**
Village clinic		1.9, (0.6–3.2)	NA	
Diagnosis and treatment: n (% or SD)				
Finger prick	165 (41.1)	96 (49.0)	69 (33.7)	0.002*
Drugs prescribed	191 (47.6)	109 (57.1)	82 (42.9)	0.191
AL	105 (26.2)	69 (35.2)	36 (17.6)	< 0.001*
Other anti-malarial drug^c^	22 (5.5)	15 (3.6)	7 (1.0)	0.10
Antibiotics	29 (7.2)	18 (9.2)	11 (5.4)	0.401
Paid for treatment	86 (21.4)	41 (20.9)	45 (22.0)	0.809
Amount in MK (SD)		450 (186–714)	426 (297–555)	0.863

*AL* artemether–lumefantrine, *AMD* anti-malarial drug, *MK* Malawi Kwacha, *PHC* primary health care facility (provide diagnosis and treatment for malaria)

* χ^2^; ** t test. Italic *p* values are statistically significant

^a^Children aged 6–59 months and women aged 15–49 months

^b^8 children were seen at village clinic

^c^Other anti-malarial drugs were sulfadoxine–pyrimethamine, quinine and artesunate–amodiaquine (second line treatment of uncomplicated malaria)

### IPTp-SP

**Fig. 1:**
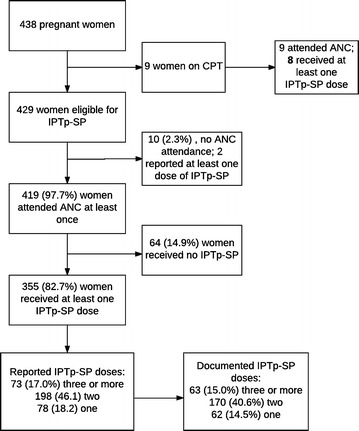
IPTp-SP in pregnancy. The proportion of women *reporting 3 or more doses* of IPTp-SP (17.0%) and those with a *documented IPTp*-*SP of 3 or more doses* (15.0%) were not different (*p* = 0.350). *ANC* antenatal care, *CPT* cotrimoxazole prophylactic therapy, *IPTp-SP* intermittent preventive therapy in pregnancy with sulfadoxine–pyrimethamine

The total number of women who had given birth within the previous 2 years was 438 (Fig. 1) of whom 429 were eligible to receive IPTp-SP. Of the eligible women, antenatal care (ANC) attendance was high, although 14.9% did not receive SP despite attending ANC. The recommended minimum three doses of IPTp-SP was documented in 15.0% of the women although 17.0% had reported completing the dose completion; there was no difference (statistically) between the two proportions (*p* = 0.350).

### ITN use, fever and care-seeking for fever by sampling round

**Fig. 2:**
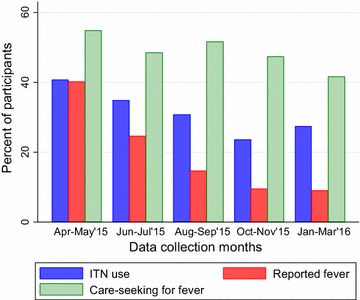
ITN use, fever and care-seeking for fever by round. There was a difference by sampling round in ITN use (*p* < 0.001) and reported fever (*p* < 0.001). There was no difference in treatment seeking for fever by sampling round (*p* = 0.612)

Both ITN use and reported fever decreased steadily from April/May 2015 (after the rainy season) to October/November (Fig. 2). Care-seeking for fever remained between 40 and 55% for those with a reported fever.

### Factors associated with ITN use, care-seeking and IPTp-SP utilization

Table 3 presents factors associated with the three primary outcomes. Wealthier households were more likely to use ITNs than less wealthy ones. Compared to the first sampling round in April/May 2015, ITN use was lower in subsequent months. Children and wealthier households were more likely to seek care than women and poorer households, respectively. With regard to completion of IPTp-SP utilization in prior pregnancy, there were no statistically significant factors associated with the outcome.

**Table 3:** Factors associated with ITN use, health care-seeking and IPTp-SP in rural Chikwawa

	aOR (95% CI)
ITN use	
Participants	
Children	Ref
Women	0.84 (0.69–1.00)
Wealth	
Lowest	Ref
Second	*1.90 (1.36–2.64)*
Middle	1.28 (0.93–1.77)
Fourth	*1.74 (1.26–2.41)*
Top	
Sampling round	
April–May’15	*Ref*
June–July’15	*0.75 (0.57–0.98)*
August–September’15	*0.63 (0.48–0.84)*
October–November’15	*0.44 (0.32–0.58)*
January–March’16	*0.53 (0.40–0.71)*
Household size	0.98 (0.94–1.03)
Sought treatment	
Participants	
Children	Ref
Women	*0.52 (0.34–0.79)*
Wealth	
Lowest	Ref
Second	0.85 (0.41–1.76)
Middle	0.87 (0.43–1.75)
Fourth	1.29 (0.64–2.61)
Top	1.67 (0.84–3.32)
Sampling round	
April–May’15	Ref
June–July’15	1.01 (0.60–1.70)
August–September’15	0.87 (0.47–1.62)
October–November’15	0.76 (0.35–1.64)
January–March’16	0.80 (0.37–1.73)
Distance to health facility (km)	1.06 (0.95–1.19)
Total people in household	1.04 (0.92–1.17)
3 or more IPTp-SP doses	
Age	0.997 (0.99–1.00)
Education category	
None	Ref
Some primary	0.54 (0.28–1.02)
Completed primary or some secondary	0.90 (0.34–2.40)
Completed secondary	0.47 (0.05–4.63)
Wealth quintile	
Lowest	Ref.
Second	1.75 (0.70–4.41)
Middle	1.26 (0.50–3.17)
Fourth	1.58 (0.64–3.90)
Top	1.47 (0.53–4.09)
Distance to health facility (km)	0.93 (0.79–1.09)

Italic confidence intervals are statistically significant

*aOR* adjusted odds ratio, *CI* confidence interval, *IPTp-SP* intermittent preventive treatment in pregnancy with sulfadoxine pyrimethamine

## Discussion

The study findings suggest suboptimal utilization of malaria control interventions in the study area. ITN ownership and usage, prompt care-seeking for diagnosis and effective treatment of malaria, and IPTp-SP rates in these communities are lower than both national estimates, and universal coverage targets. This highlights that national coverage aggregations mask substantial inequities in intervention coverage [10] and are not ideal for identifying and targeting high burden and low intervention marginalized communities. District-based and fine-scale surveillance may improve identification of these marginalized communities. The Malawi National Malaria Control Programme conducted free nationwide ITN distribution campaign in April 2016, and increased availability of free diagnosis and treatment of malaria in both public and private health facilities. Recently, provision of uncomplicated malaria diagnosis and treatment for children below 5 years old by community health workers has increased availability and access in remote communities. MMP also implemented a community-led behaviour change programme to improve care-seeking and ITN use. An evaluation of access and utilization of the interventions following their scale up and MMP community-led intervention will be published.

There were delays in care-seeking for fever for both children and women, with some not seeking care at all (Table 2), similar to other studies in Africa [24]. Only a small proportion of participants (19.5%) sought care within 24 h of symptom onset. Untreated symptomatic or asymptomatic cases are parasite reservoirs for continued transmission [25]. Although distance to a health facility has been reported in the same area to affect care-seeking [22, 23], distance was not a significant factor in the current study. Distance to facility may have been an important factor in previous reported studies which had a wider variation in distance (communities located 8 km or more were compared with those close to the hospital) than the current study (less than 3 km on average). In the current study, an estimate of the distance between the household and a primary health facility was a Euclidean distance calculated based on GPS locations. This estimation method may not reflect the true distance of the actual path between the household and health facility which is determined by geographical (i.e. terrain) and man-made barriers. The lack or delay in accessing diagnosis and treatment of fever may be more related to socio-cultural factors which were not investigated in this quantitative analysis. MMP is currently conducting qualitative research and implementing a malaria behaviour change communication (BCC) strategy [26] within the community to understand and improve, respectively, socio-cultural factors affecting care-seeking.

For ITN ownership and usage, the findings suggest national ITN coverage estimates, which are reported every 2 years, underrepresent rural communities. Although this study was conducted in one rural community, household ITN ownership (35.3%) and under five ITN usage (33.5%) are lower than the 2014 MMIS estimates of 70.2 and 67.1%, respectively [13]. The national surveys use self-reported responses to estimate ITN usage, similar to the current study. Low ITN ownership and usage highlight the gap in ITN coverage for poor communities similar to findings in Uganda and Tanzania [27, 28]. Even within these rural communities, diminishing wealth was associated with lower ITN usage, highlighting health inequity for the poor [29]. Household ITN ownership was previously reported to be associated with lower odds of parasitaemia in children in this community [17], meaning that the children in households without ITNs are not only at higher risk of malaria infection, but also serve as a source of malaria parasites to surrounding communities. These poor households and communities are not identified in national surveys for targeted interventions. Evidence for improving household ownership of ITN through mass distribution campaigns has yielded mixed results in sub-Saharan Africa; in some countries, equity between the relatively wealthier and the less wealthy has increased, while in others, it decreased [30–32]. Deliberately addressing inequity and specifically developing strategies aimed at improving community socio-economic status may improve malaria control for poor communities [33]. For instance, fine-scale mapping of malaria burden and intervention coverage can potentially assist identifying and targeting marginalized communities. Surveillance using district-based health teams may also increase fine scaling mapping and identifying such communities. Most rural areas have community health workers who can be utilized for this role.

The proportion of women confirmed to have taken the recommended three or more doses of IPTp-SP (15.0%) was lower than those who took two doses (40.6%) and comparable to 2014 MMIS estimates (12.6%). The proportion is significantly lower than 31% reported in World Malaria Report 2016 for 20 African countries [3]. Three SP doses are associated with higher neonatal birth weights, reduced risk of low birth weight and reduced risk of placental malaria, compared to two doses [34, 35]. No quantitative variables were significantly (statistically) associated with completion of IPTp-SP doses. The lack of significant variation in the predictors and sparse data, account for this finding.

The implementation of malaria interventions through the health system were suboptimal. Although reported utilization of ANC services was high, the supply of IPTp-SP was inadequate (Fig. 1), as 14.9% of women who attended ANC did not receive it. This discrepant use of IPTp-SP needs further investigation from both health system and patient perspective. For those who sought care for fever, the number who had a finger prick for malaria test was 41%, far below the universal access to malaria diagnosis target. Health system barriers for IPTp-SP and diagnosis and treatment of malaria were not investigated in this study, although in other studies, these affect rural areas disproportionately to urban areas. Barriers include availability of medical supplies and understaffing, [14] as most health care workers do not want to work in remote areas.

The survey relied on interviewee responses, which are standard in most national household surveys. However, documented information on IPTp-SP use in health passports was used as an additional more reliable source of information. Reported IPTp-SP and ITN use during surveys are susceptible to recall and social desirability bias [36–38]. For IPTp-SP, there was no difference (statistically) between interviewee response and documented information, suggesting that interviewee responses were reliable. For ITN use, a previous study in Malawi validated the accuracy of care-giver response for evaluating ITN use in children; the study was however conducted in a community with higher ITN ownership (following an ITN distribution campaign) than the current study and also reported a low accuracy for people who reported no ITN use [39].

Although this study was conducted in one rural setting, the situation may not be unique to this community. More fine-scale studies need to be conducted focusing specifically on rural regions, where the majority of people in developing countries reside, to improve malaria control.

## Conclusions

Overall, the results highlight the need to investigate and address health system and community barriers to utilization of malaria diagnosis and treatment and IPTp-SP programmes in rural communities. The burden of malaria will remain high in rural communities if deliberate efforts are not taken to identify and address community utilization and disparities in intervention coverage, and to improve quality of interventions. Efforts should be scaled up to include remote communities where health systems are poor, surveillance is low and social and cultural factors may play an important role in determining utilization. The use of district-based household surveillance to monitor coverage of interventions may improve access and utilization through identification and targeting of communities requiring scale up of interventions.

## Abbreviations

ACT: artemisinin-based combination therapy
AL: artemether–lumefantrine
AMD: anti-malarial drug
ANC: antenatal care
aOR: adjusted odds ratio
ASAQ: artesunate–amodiaquine
BCC: behaviour communication change
CI: confidence interval
CPT: cotrimoxazole preventive therapy
GPS: Global Positioning System
HRP: histidine-rich protein
IPTp-SP: intermittent preventive therapy in pregnancy with sulfadoxine–pyrimethamine
ITN: insecticide-treated bed net
IQR: interquartile range
MMIS: Malawi Malaria Indicator Survey
MIS: Malaria Indicator Survey
MMP: Majete Malaria Project
MWR: Majete Wildlife Reserve
PHC: primary health care facility
rMIS: rolling Malaria Indicator Survey
SD: standard deviation
SP: sulfadoxine–pyrimethamine
